# Factors associated with oropharyngeal dysphagia in individuals with cardiovascular disease and COVID-19

**DOI:** 10.1590/2317-1782/20242022112en

**Published:** 2024-08-19

**Authors:** Tatiana Magalhães de Almeida, Raquel Gama Fernandes, Vitor Della Rovere Binhardi, João Italo Dias França, Daniel Magnoni, Roberta Gonçalves da Silva

**Affiliations:** 1 Instituto Dante Pazzanese de Cardiologia - São Paulo (SP), Brasil.; 2 Universidade Estadual Paulista – UNESP - Marilia (SP), Brasil.

**Keywords:** Cardiovascular Diseases, Swallowing Disorders, Intratracheal Intubation, Coronavirus Infections, Aging

## Abstract

**Purpose:**

Oropharyngeal dysphagia (OD) is one of the possible outcomes in patients hospitalized with COVID-19 and also in the population hospitalized for the treatment of cardiovascular disease. Thus, knowing the predictive risk factors for OD may help with referral and early intervention. This study aimed to verify the association of different factors with OD in hospitalized individuals with cardiovascular disease and COVID-19.

**Methods:**

Cross-sectional clinical study approved by the Research Ethics Committee (4,521,771). Clinical evaluation of swallowing was carried out in 72 adult patients with cardiovascular disease and COVID-19 hospitalized from April to September 2020. Individuals under 18 years of age and without previous cardiovascular disease were excluded. The presence of general clinical and/or neurological complications, pronation, stay in the intensive care unit (ICU), orotracheal intubation (OTI), tracheostomy tube, oxygen support and age were considered as predictive risk factors for oropharyngeal dysphagia. Fisher's exact test, Mann Whitney test and logistic regression model were used for analysis.

**Results:**

General clinical complications (p=0.001), pronation (p=0.003), ICU stay (p=0.043), in addition to the need for oxygen supplementation (p=0.023) and age (p= 0 .037) were statistically significant factors associated. The pronation (0.013) and age (0.038) were independently associated with dysphagia. OTI (p=0.208), tracheostomy (p=0.707) and the presence of previous cerebrovascular accidents (p=0.493) were not statistically significant.

**Conclusion:**

In this study, age and prone position were factors independently associated with oropharyngeal dysphagia, complications such as the need for oxygen supplementation, in addition to the need for ICU admission, were also associated factors in the population.

## INTRODUCTION

In the world health context we are faced with the COVID-19 pandemic, named SARS-CoV-2 by the World Health Organization WHO^(1)^. In face of the unprecedented situation, and with the outbreak already installed in the world, several basic diseases were considered risk factors to increase the lethality degree of COVID-19, and among them cardiovascular diseases.

It is known that the patient with cardiovascular disease may present immunological impairments and a chronic inflammatory state that may favor contagion and worsening of the disease^(2)^. Recent COVID-19 studies have shown that individuals with associated comorbidities especially those with hypertension, coronary heart disease, or diabetes are at higher risk of contamination and are more likely to develop severe symptoms^(2,3)^.

Oropharyngeal dysphagia is a condition that has been cited in hospitalized patients with COVID-19^(4-6)^. The causes are multifactorial and the need for prolonged mechanical ventilation is evidenced, a condition in itself already considered an isolated risk factor for oropharyngeal dysphagia in other health conditions, since it may compromise the sensitivity, mobility and strength of the structures involved in swallowing, the incidence being variable and co-dependent on several other factors, and may reach 1/3 after extubation^(7)^.

However, it is important to consider its particularities as this patient may also present other potential risk factors for dysphagia associated, such as reduced pulmonary capacity, shortened and weakened breathing that may compromise the coordination between breathing and swallowing^(6,8)^, as well as other aspects such as advanced age and history of other associated comorbidities, and COVID-19 may also lead to peripheral and central swallowing damage^(9,10)^. Furthermore, the population hospitalized for treatment of cardiovascular disease also presents risk factors for dysphagia, where prolonged intubation stands out, in addition to advanced age, previous or recent neurological deficits such as stroke and nutritional decline^(11)^.

Thus, in the face of this new disease, studies on the performance of oropharyngeal swallowing in patients with COVID-19 and previous cardiovascular disease are considered fundamental to verify which factors aggravate the risk of dysphagia in this population. This study aimed to associate different factors with oropharyngeal dysphagia in hospitalized individuals with cardiovascular disease and COVID-19.

## METHOD

### Population

Eighty patients with cardiovascular disease and COVID-19 confirmed by reverse transcriptase-polymerase chain reaction (RT-PCR) test were admitted to a public referral cardiology hospital from April to September 2020 were referred, evaluated and followed by the speech language therapy team. Of these, 72 patients were included, 62.5% (45 patients) being male and 37.5% (27) female, with a mean age of 69.22 years. Patients under the age of 18 years, with no previous cardiovascular disease, and in whom the patients and/or their families did not authorize the use of protocol data for the research were excluded.

Among the comorbidities associated with cardiovascular disease, the individuals had hypertension, dyslipidemia, stroke, heart failure, coronary syndrome, coronary artery disease, atrial fibrillation, arrhythmia, and others.

### Method

Retrospective clinical study approved by the Research Ethics Committee under protocol 4.521. 771. Oropharyngeal swallowing was evaluated using the clinical protocol presents data on identification, hospitalization diagnosis, respiratory status, feeding route, indirect and direct evaluation of swallowing with different food consistencies and volumes based in the literature^(12,13)^.The presence of oropharyngeal dysphagia was diagnosed when were observed impairment in the oral phase (deficit in lip capture and sealing, impairment in oral transit time, considering the time between food intake and the beginning of the pharyngeal response), and/or pharyngeal phases of swallowing,(reduced laryngeal elevation, presence of incomplete swallowing, absence of swallowing, multiple swallows and clinical signs suggesting penetration and/or aspiration like coughing, choking, throat clearing, wet voice) .The oropharyngeal dysphagia was classified as mild, moderate or severe based on parameters indicated in the literature^(14)^.

Were considered predictive risk factors for oropharyngeal dysphagia the variables clinical complications related to COVID-19 and classified in the service by the medical team (renal failure, bleeding, acute myocardial infarction myocarditis, arrhythmia, pulmonary thromboembolism) and separately the need pronation, orotracheal intubation, presence of tracheostomy tube, need for oxygen support, stroke, and age.

For statistical analysis, univariate and multivariate analysis was performed. The variables with clinical significance considered in the univariate analysis were: clinical complications, pronation, ICU admission, orotracheal intubation, presence of tracheostomy tube, oxygen support, stroke, and age. Age was placed continuously in the models, while for qualitative variables the absence of the condition was used as a reference for calculations. Despite the clinical significance, the variable stroke and general complications were removed from the logistic regression analysis, considering that 100 percent of patients with dysphagia had some general complication and all patients with stroke had some degree of dysphagia, therefore they did not present what occurs in the absence from them.

A multivariate logistic model was also carried out to identify independently associated factors, and the results of the logistic regression were represented by adjusted odds ratios (OR) with 95% confidence intervals.

Fisher's exact test and Mann Whitney were used for the statistical analysis considering a significance level of 0.05.

To assess whether the logistic regression model is truly effective, the Hosmer-Lemeshow Test was performed, aiming to assess the quality of the model fit, whose p-value was equal to 0.123, indicating a good fit of the model.

## RESULTS

Of the 72 (100%) individuals evaluated, 37 (51.4%) presented some degree of oropharyngeal dysphagia in the first clinical evaluation of swallowing. These 37 (100%) dysphagic patients, 22 (59.43%) had mild, 12 (32.43%) moderate e 3 (8.10%) severe (Table 1).

**Table 1:** Frequency of oropharyngeal dysphagia by degree of severity of swallowing disorder

	Number (N)	Percentage (%)
Oropharyngeal dysphagia	37	100%
Mild	22	59.45%
Moderate	12	32.43%
Severe	3	8.10%

N=Number of individuals

The Table 2 shows the association between the variables studied in individuals with and without dysphagia.

**Table 2:** Association of variables in individuals with and without dysphagia

**Variable**	**Without Dysphagia**	**With Dysphagia**	**P-value**
General complications, n (%)	29 (82.86%)	37 (100%)	0.010
Age, mean (SD)	66.94 (8.53)	71.38 (10.25)	0.037
Pronation, n (%)	2 (5.71%)	13 (35.14%)	0.003
Oxygen support, n (%)	23 (65.71%)	33 (89.19%)	0.023
ICU, n (%)	24 (68.57%)	33 (89.19%)	0.043
Orotracheal intubation, n (%)	21 (60%)	28 (75.68%)	0.208
Tracheostomy, n (%)	4 (11.43%)	3 (8.11%)	0.707
Stroke, n (%)	0 (0%)	2 (5.41%)	0.493

SD: standard deviation; n: number; ICU: intensive care unit

The Table 3 represents the univariate (crude) and multivariate (adjust) logistic model to check which variables are independently associated with oropharyngeal dysphagia in the population studied represented by adjusted odds ratios

**Table 3:** Odds ratio (OR) for oropharyngeal dysphagia according to variables

**Variable**	**Category**	**Crude**	**Adjust**	
		OR (95%CI)	OR (95%CI)	P-value
Age		1.05 (0.999 – 1.109)	1.07 (1.004 – 1.136)	0.038
Pronation	Yes	8.94 (1.843 – 43.34)	8.91 (1.578 – 50.355)	0.013
	No (Ref.)	1.00	1.00	
Oxygen support	Yes	4.30 (1.233 – 15.032)	2.94 (0.69 – 12.556)	0.145
	No (Ref.)	1.00	1.00	
ICU	Yes	3.78 (1.073 – 13.322)	3.31 (0.588 – 18.631)	0.175
	No (Ref.)	1.00	1.00	
Orotracheal intubation	Yes	2.07 (0.755 – 5.698)	0.68 (0.158 – 2.97)	0.613
	No (Ref.)	1.00	1.00	
Tracheostomy	Yes	0.68 (0.142 – 3.300)	0.47 (0.07 – 3.157)	0.438
	No (Ref.)	1.00	1.00	

## DISCUSSION

Oropharyngeal dysphagia has been described in the population with COVID-19^(4-6)^ and may be associated with the symptoms of the disease, possible complications of the condition, and also with the procedures involved in the treatment. Because it is a new condition, the frequency of dysphagia, as well as the possible causes, are still poorly described. In the current study, 51.4% of the patients referred for speech evaluation and follow-up had some degree of oropharyngeal dysphagia. These patients were referred for Speech Language Pathologist evaluation by the medical team, which considered as predictive risk factors for dysphagia the markers previously used at the institution and among these we highlight the need for prolonged orotracheal intubation, tracheostomy tube, signs and symptoms suggestive of dysphagia, as well as patient complaints regarding swallowing.

A recent study evaluating the swallowing of patients with COVID-19, but did not specify comorbidities or other associated diseases, also utilized the clinical evaluation as a method of investigation with evaluation of oral motor function, vocal quality and biomechanics of swallowing with multiple food consistencies, and considered in the risk screening for referral for specialized evaluation the presence of coughing during or after swallowing, pain or swallowing difficulty, reduced oral intake or aspiration events identified in the X-ray. The study reported that 208 patients out of 720 inpatients were referred for specific evaluation based on the above criteria, but did not clarify the definition of the outcome for dysphagia, reporting only data on release and/or contraindication of oral pathway, and more than 60% of the population admitted to the intensive care unit (ICU) had contraindication for oral pathway after the first evaluation^(15)^. 

A similar study that used only a multidimensional tool designed to measure both required supervision and dietary levels after the initial assessment found that 19.8% of patients had a deficit in swallowing safety and 53.5% were classified in levels 4 and 5 where swallowing is classified as safe, but required dietary restriction or use of compensatory strategies^(5)^. The population consisted of 101 patients with prolonged intubation longer than 48 hours who were stable after extubation. There is no clear data regarding the classification of swallowing function and the comorbidities associated with COVID-19 were not reported, it is noteworthy that the present study population have cardiovascular disease associated with COVID-19 which may have impacted the frequency of this disorder since this condition also presents risk factors for dysphagia^(11)^.

Based on our expertise in caring for patients with oropharyngeal dysphagia, it is possible to understand that COVID-19 is not only a respiratory disease, but a systemic one; therefore, the potential causes of dysphagia in this population should not be limited to the disease alone, but to the entire clinical condition involved in the patient's hospitalization. The risk factors that permeate this condition are still unclear in the literature.

The first reports of the risk for dysphagia after COVID-19 were related to the need for prolonged orotracheal intubation^(4)^, being this condition already considered a risk in other contexts with variable incidence due to methodological differences between studies such as health conditions and different methods of swallow evaluation. It is known that intubation can lead to trauma of the oropharynx, larynx, sensory deficit leading to the risk of dysphagia. Besides mechanical and sensory alterations, global neuromuscular weakness with impact on the muscles involved in the swallowing and breathing function is also a possible cause. This may be caused by disuse of the muscles, besides prolonged periods under the effect of sedatives and/or after the use of neuromuscular blockers. The need for medications with sedative or neurotrophic effects can affect swallowing not only with an impact on the neuromuscular junction, but also with an impact on the level of consciousness^(16-19)^.

In the current study there was no statistical significance between the presence of orotracheal intubation and oropharyngeal dysphagia (p=0.208) (p=0.613), and intubated patients were not more likely to have dysphagia (OR:0.68), more than 60% of the patients in the sample required orotracheal intubation, the prolonged intubation factor is already a risk factor considered in the service for speech therapy referrals, in addition to being a common condition in COVID-19 factors that may have led to the large number of patients in this condition, however, when analyzing the group with dysphagia, it was observed that more than 75% of the patients required orotracheal intubation and in patients without dysphagia 60%, an aspect that may clinically justify the impact of intubation on swallowing. The need for a tracheostomy tube was also not statistically significant, but the sample was only 7 patients. We believe that the significance was influenced by the sample being predominantly of patients already intubated and due to the small percentage of patients who needed the tracheostomy tube.

In the present study the risk factors associated with dysphagia were related to the severity of the overall clinical picture, and statistical significance was observed between the complications of the picture considered by the clinical staff of the institution and dysphagia (p=0.010), 100% of patients with some degree of dysphagia had some complication. Other complications were analyzed in isolation because they are already known to be a risk for dysphagia in other contexts as the need for admission to the ICU (p=0.043). On the other hand, this group of patients is 3.31 times more likely to have dysphagia than patients who did not require ICU admission. We know that dysphagia is a common condition in ICU patients, and it is not only the need for mechanical ventilation that causes swallowing disorders, but also the association with neurological conditions, neuromuscular weakness and disease severity^(19-21)^. In the population studied the neurological condition involved was stroke and although there was no statistical significance, only two individuals had stroke, clinical significance was observed because all had some degree of oropharyngeal dysphagia.

The prone position has been used as adjuvant therapy to improve ventilation in patients with COVID-19 and respiratory distress syndrome^(22-24)^ was related to the condition of oropharyngeal dysphagia (p=0.003) (p=0.013). In this study, the patients who needed pronation were 8.91 times more likely to have dysphagia than patients who did not.The prone position risks are reported with this measure such as accidental extubation, facial edema, lesions in the oral cavity, pressure ulcers, difficulty in oral hygiene, and hypersalivation with risk of aspiration of microorganisms^(6,24)^. It is noteworthy that this condition is associated with orotracheal intubation and the severity of the respiratory condition, which are already risk factors for swallowing disorders.

As we know, patients with COVID-19 may develop respiratory fatigue and require supplemental oxygen, the alteration in respiratory dynamics may lead to incoordination between breathing and swallowing impacting on safety, besides interfering in the volume of oral intake with impact on nutritional aspects, being already cited as a cause of dysphagia in the population with COVID-19^(5,6).^ The need for oxygen supplementation that included the use of nasal oxygen catheter, non-invasive ventilation and high-flow catheter was related to oropharyngeal dysphagia (p=0.023) in our sample. Patients in need of oxygen support were 2.94 times more likely to have dysphagia than those who did not need it. In our practice, we observed that patients required adaptation of feeding consistency in order to favor greater safety and less fatigue thus minimizing respiratory and nutritional risks.

Moreover, the advancing age has been considered a risk factor for the severity of COVID-19, among the causes it is highlighted changes in the immune system, higher risks of associated diseases such as cardiovascular diseases, in addition to fragility^(25)^. Age also causes aging of the swallowing muscles, and the healthy elderly compensate for these losses, but the elderly with some associated disease hospitalized for treatment increase the risk of developing swallowing disorders, and the elderly in this condition should be looked at with extreme attention. In our study, age was a significant factor (p= 0.038) for dysphagia.

We understand that COVID-19 is a disease that in its severe form can lead to oropharyngeal dysphagia and that the conditions involved are not only those related to the respiratory condition, but also associated with the severity of the general clinical and neurological condition, and the presence of dysphagia is associated with worse outcomes. In the present study the swallowing disorder was related to mortality (p=0.051) and when we analyzed the hospital discharge outcome we observed that 58.7% of the patients who were discharged from the hospital had no associated dysphagia (p=0.023).

Finally, and considering that every study of swallowing in the population with COVID-19 around the world were initially carried out only with the expertise of the professional using clinical protocols not validated for this population, it is necessary to reflect on the findings regarding swallowing performance due to the accuracy of the clinical method in the general population. On the other hand, this study established clear criteria.

New studies observing the swallowing of patients with COVID-19 and with validated clinical methods and instrumental exams of swallowing biomechanics will allow us to compare inter-institutional results in their dysphagic symptomatology and degree of impairment.

## CONCLUSION

In this study, age and prone position were factors independently associated with oropharyngeal dysphagia, complications such as the need for oxygen supplementation, in addition to the need for ICU admission, were also associated factors in the population with cardiovascular disease and COVID-19.
